# Supplementary figures and images for: Dynamic Optimization with Particle Swarms (DOPS): a meta-heuristic for parameter estimation in biochemical models
Source: BMC Syst Biol. 2018 Oct 12;12:87. doi: 10.1186/s12918-018-0610-x (PMC6186122; doi:10.1186/s12918-018-0610-x)

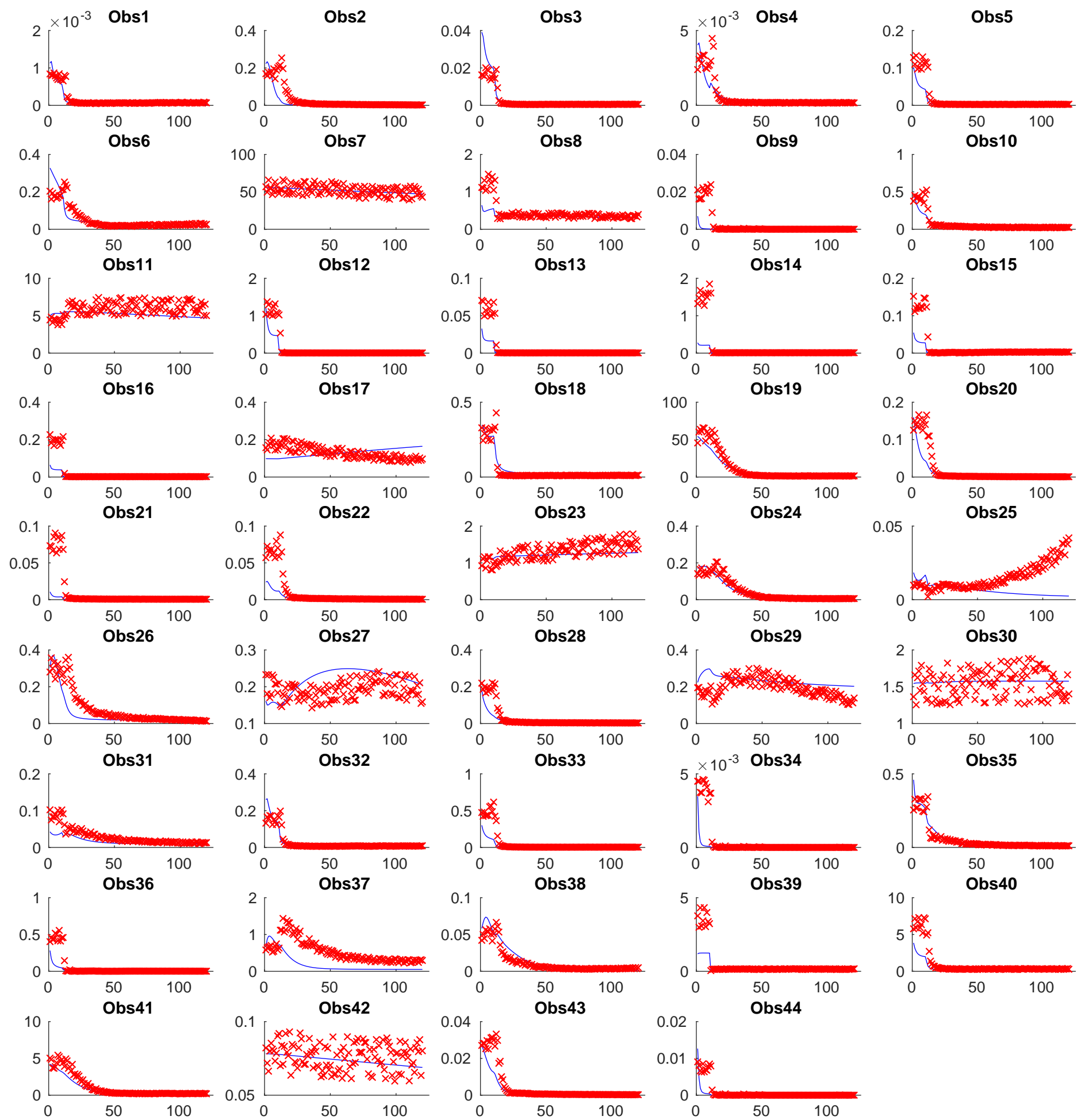

Supplement: Supplementary file 1 — Figure S1. Data fits for CHO metabolism problem. (PDF 130 kb) [file 12918_2018_610_MOESM1_ESM.pdf]

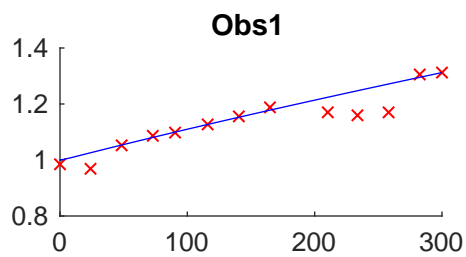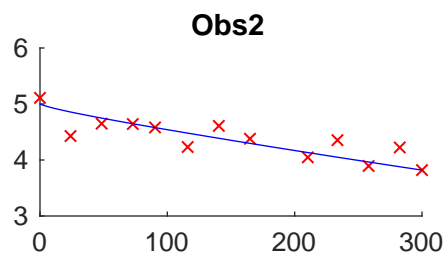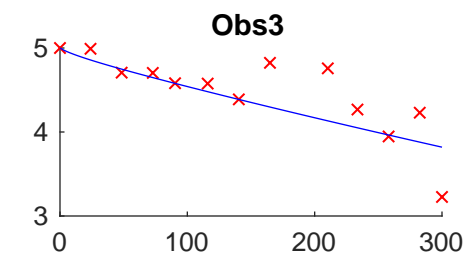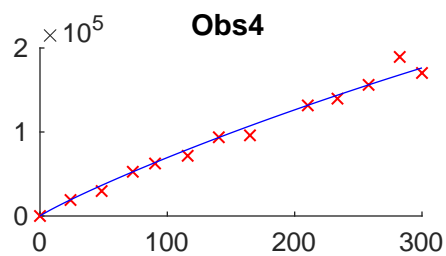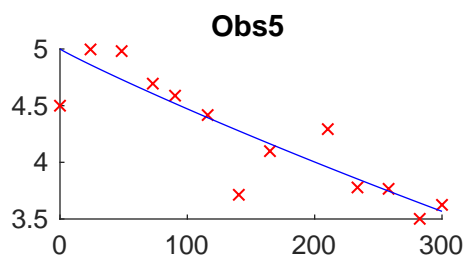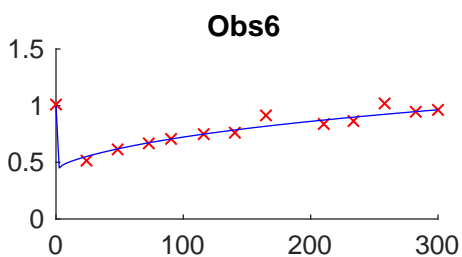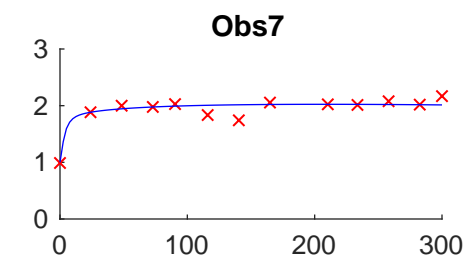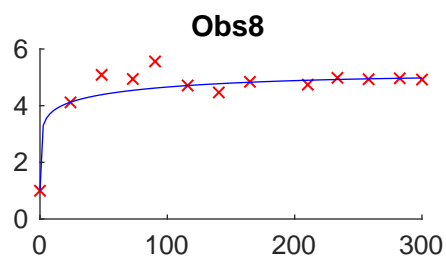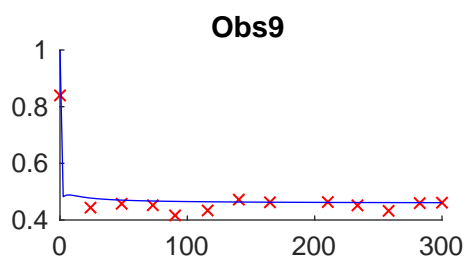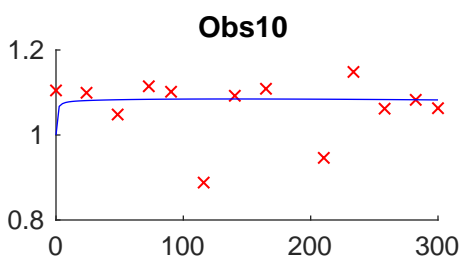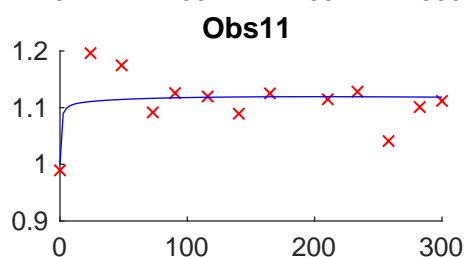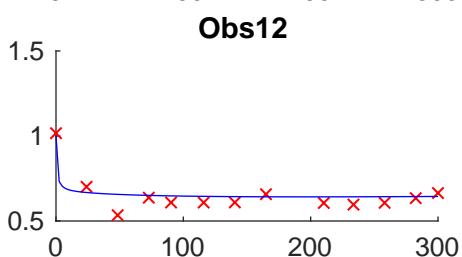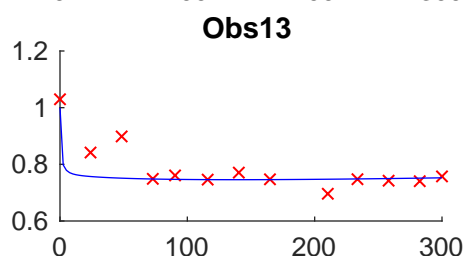

Supplement: Supplementary file 2 — Figure S2. Data fits for S.cerevisiae metabolism problem. (PDF 22 kb) [file 12918_2018_610_MOESM2_ESM.pdf]

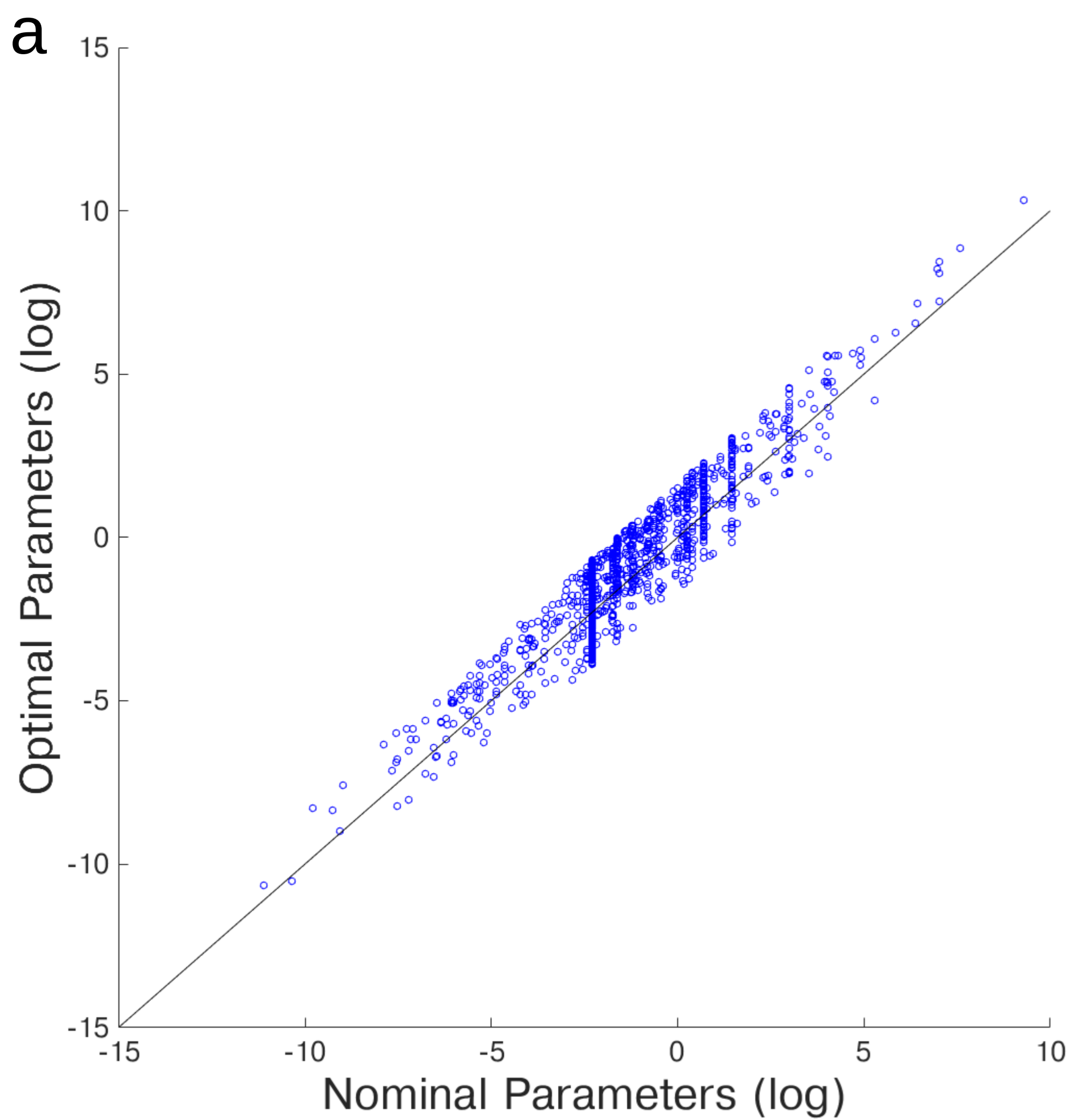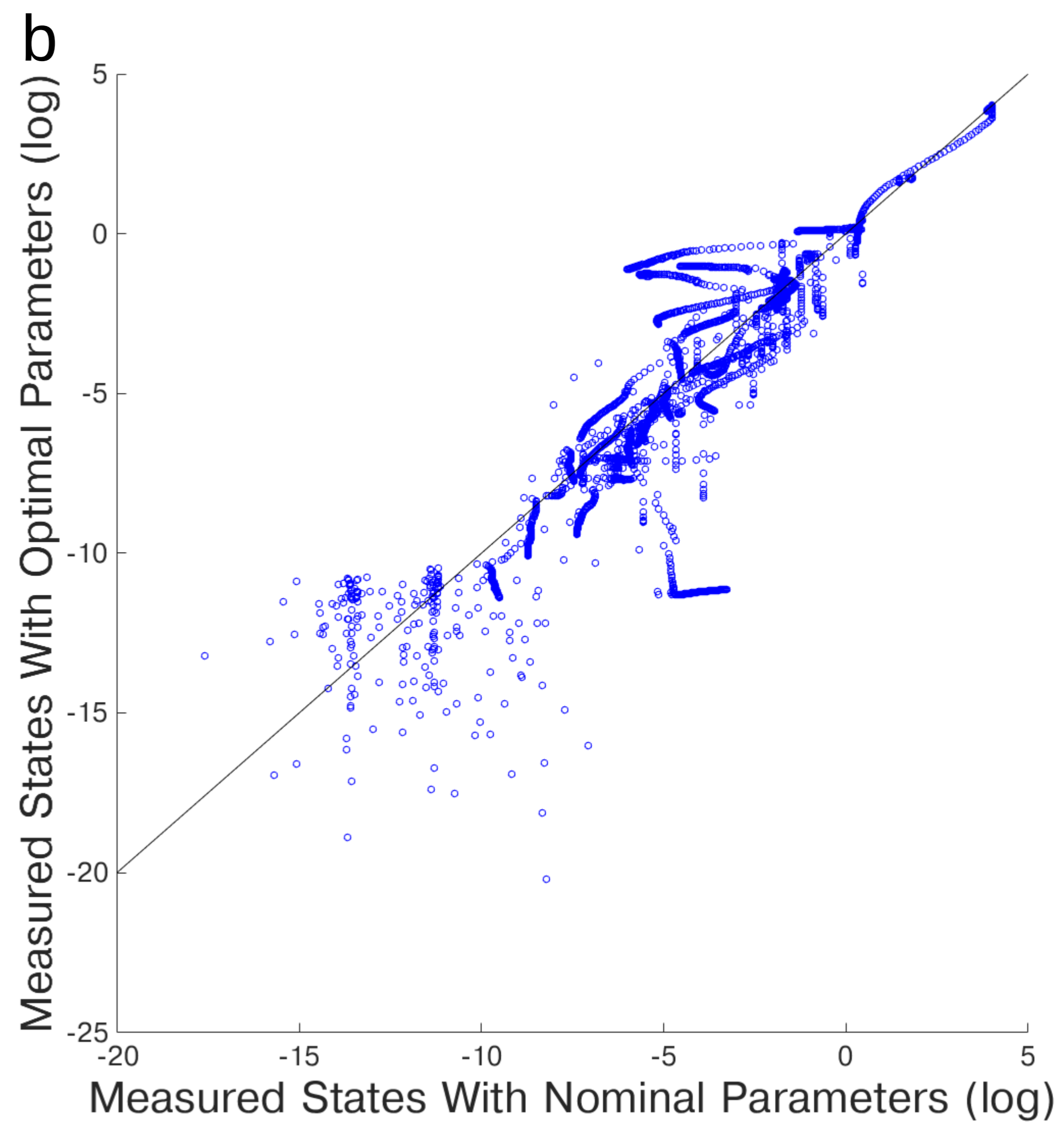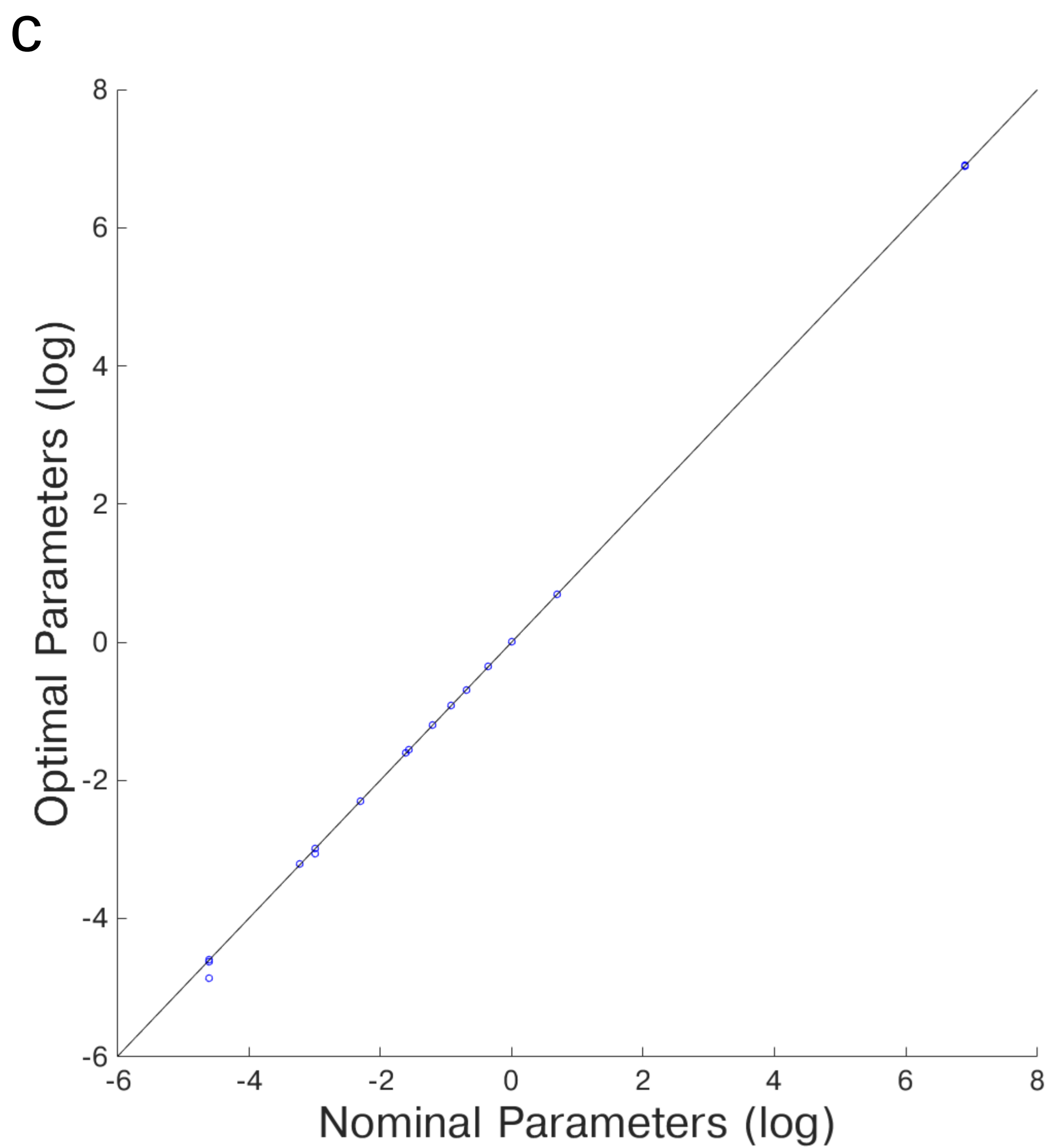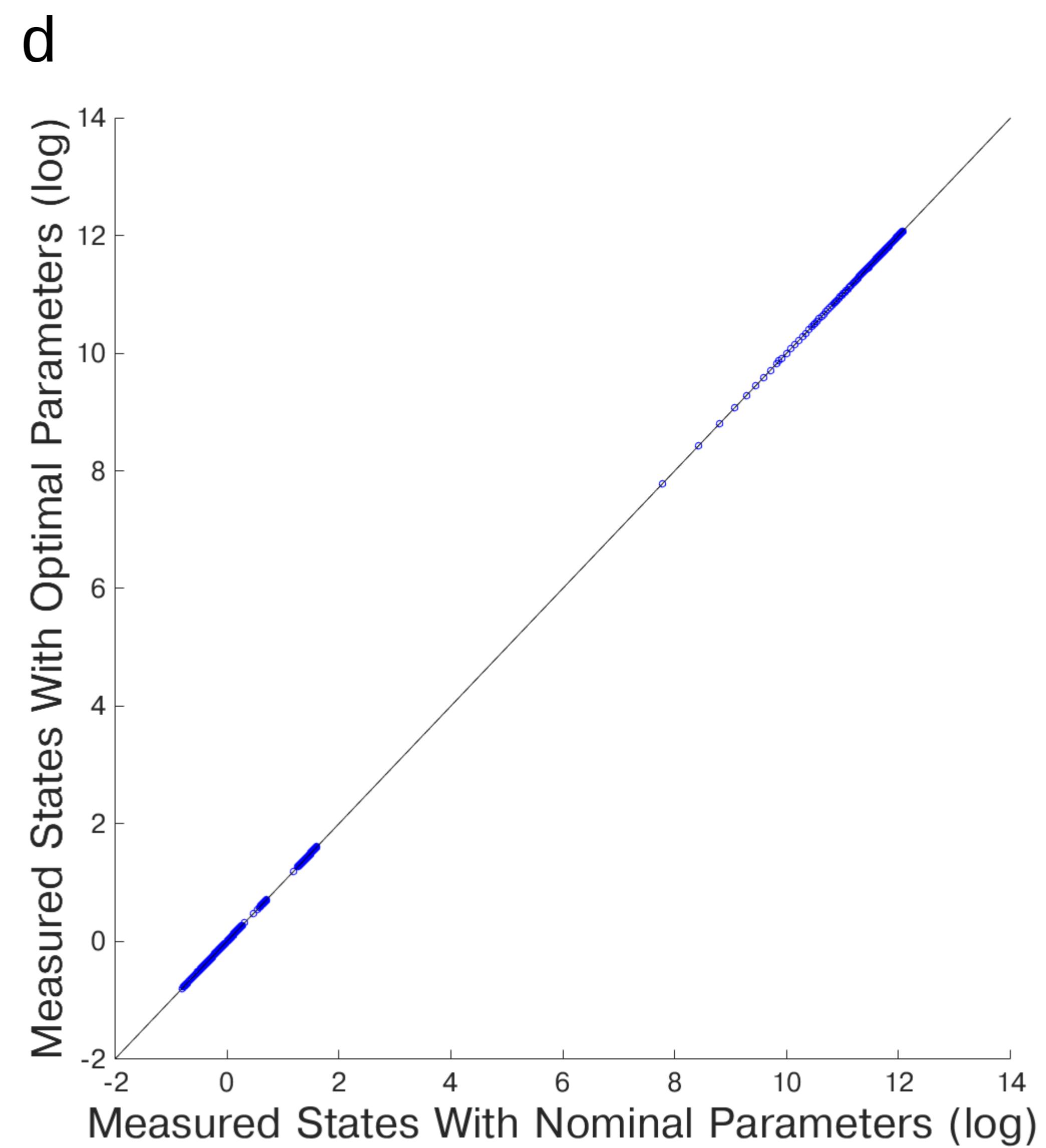

Supplement: Supplementary file 3 — Figure S3. Comparison of states and parameters. (PDF 192 kb) [file 12918_2018_610_MOESM3_ESM.pdf]

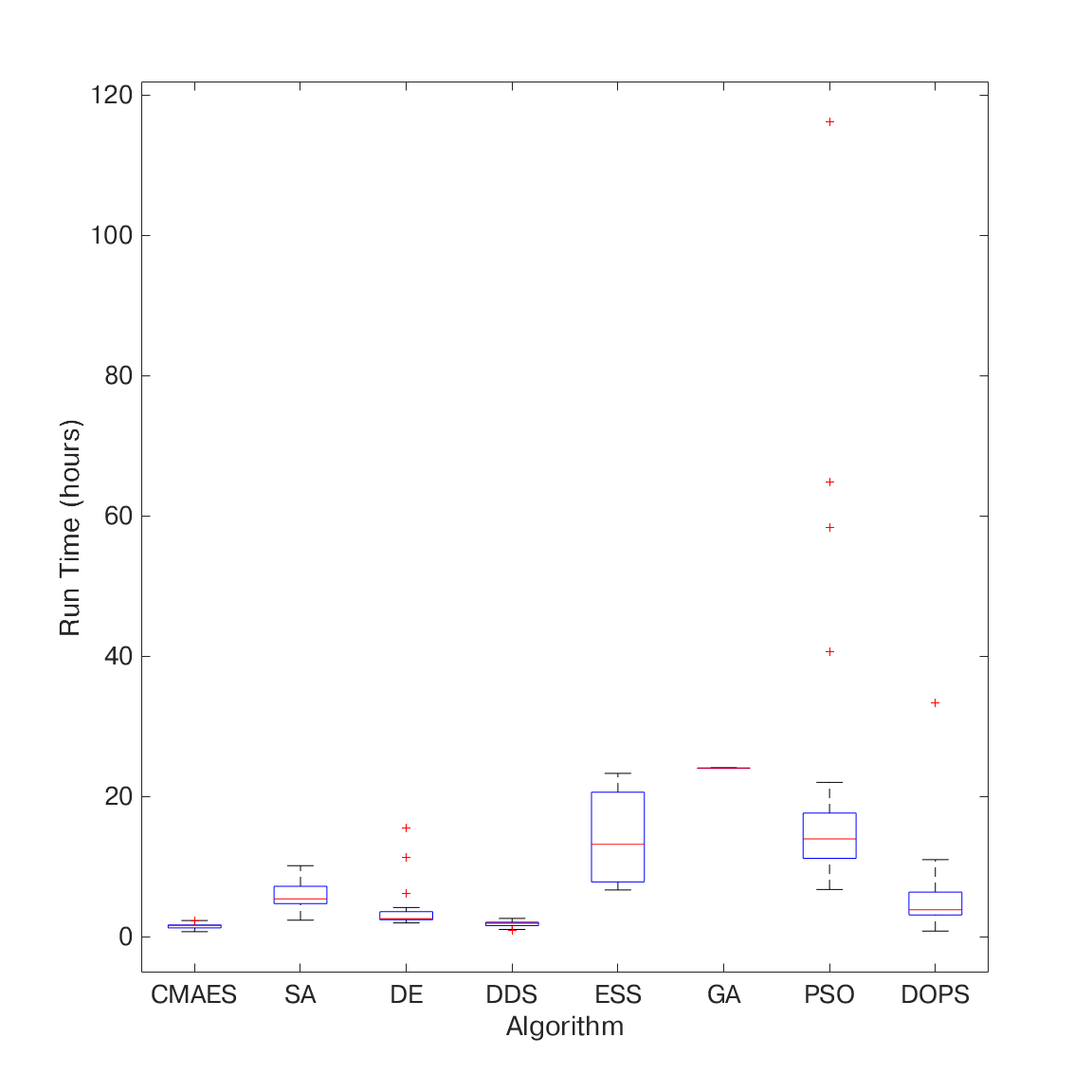

Supplement: Supplementary file 4 — Figure S4. Time Comparison. (PNG 28 kb) [file 12918_2018_610_MOESM4_ESM.png]

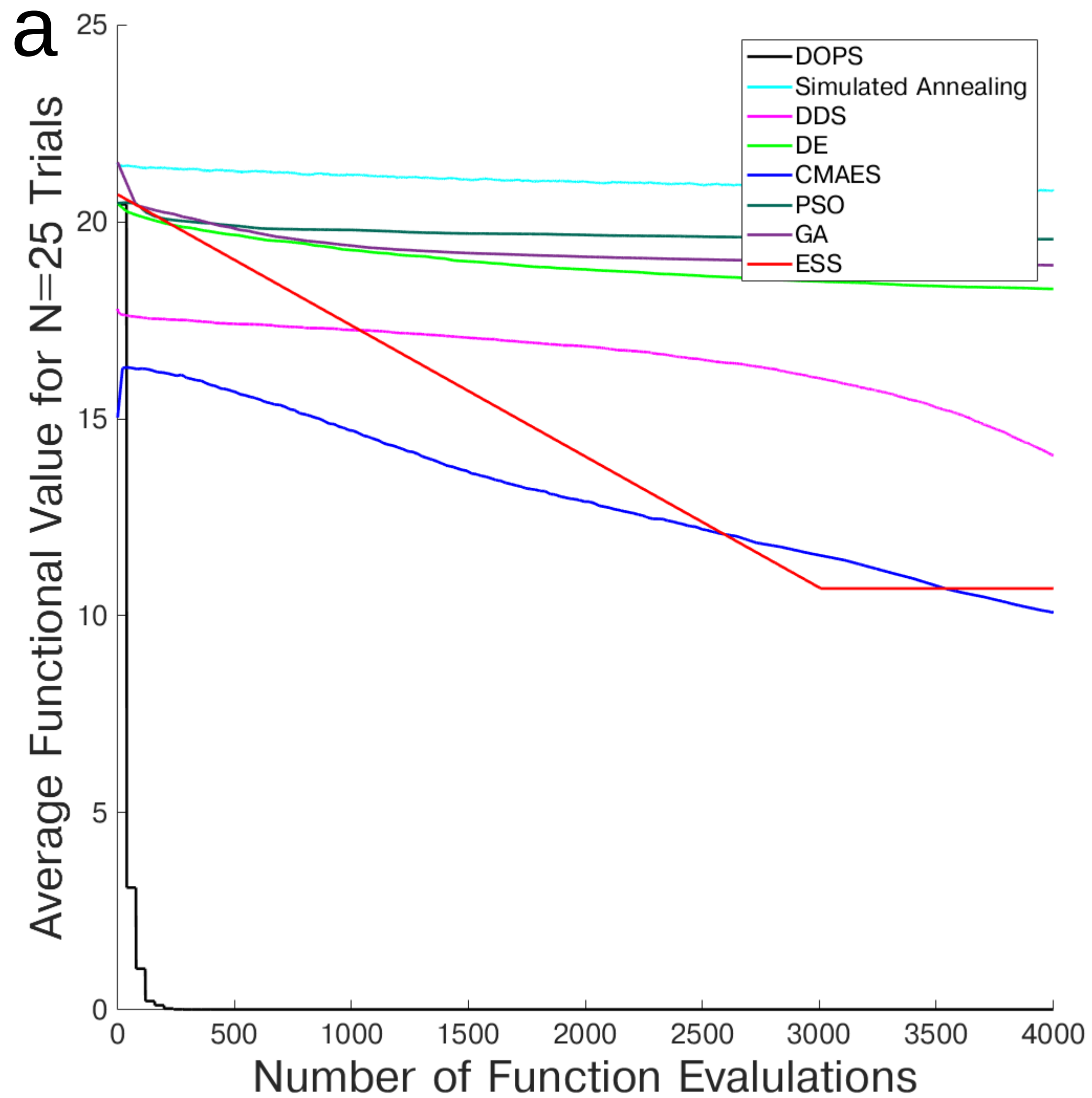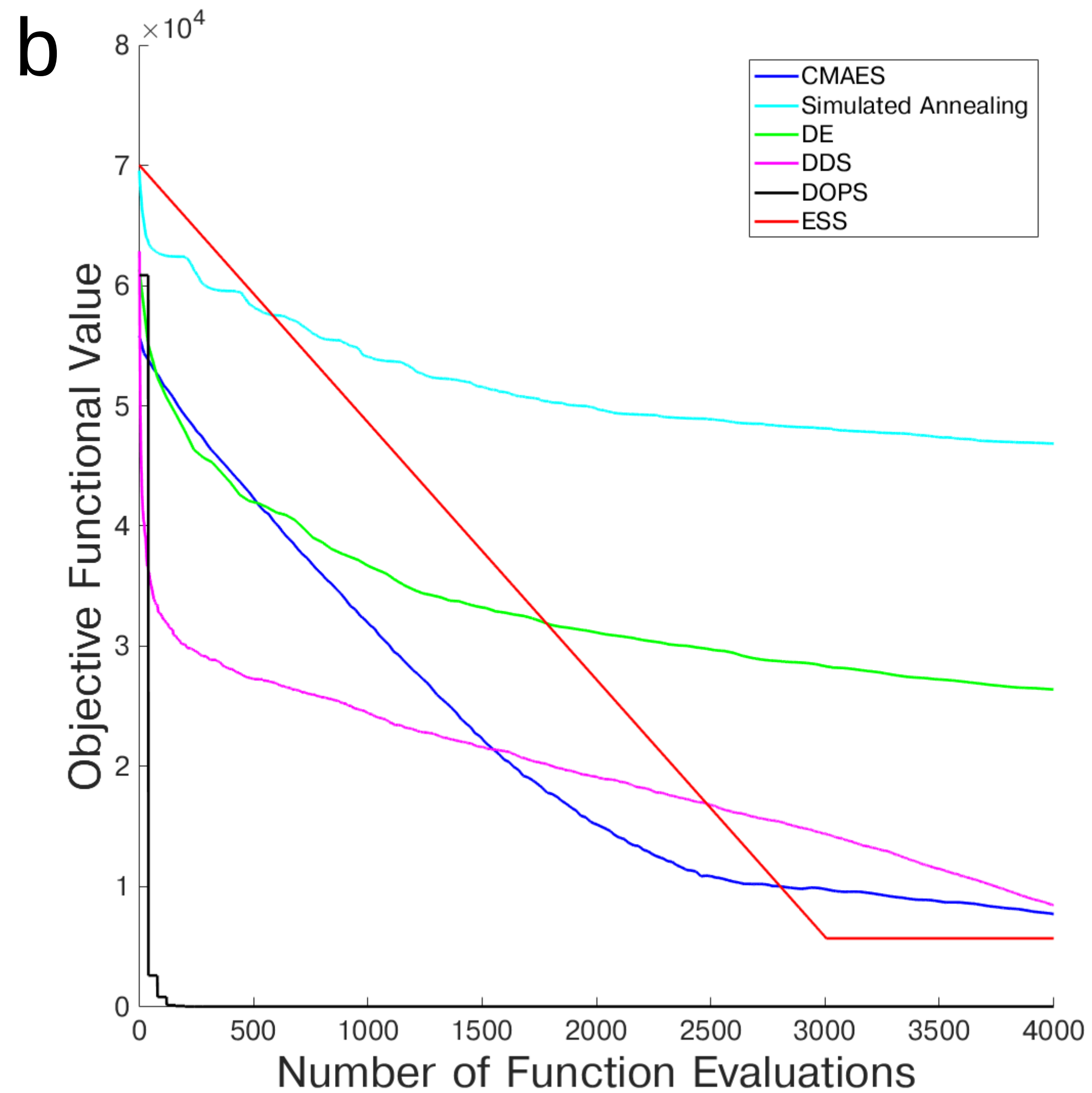

Supplement: Supplementary file 5 — Figure S5. Convergence Curves. (PDF 116 kb) [file 12918_2018_610_MOESM5_ESM.pdf]

a

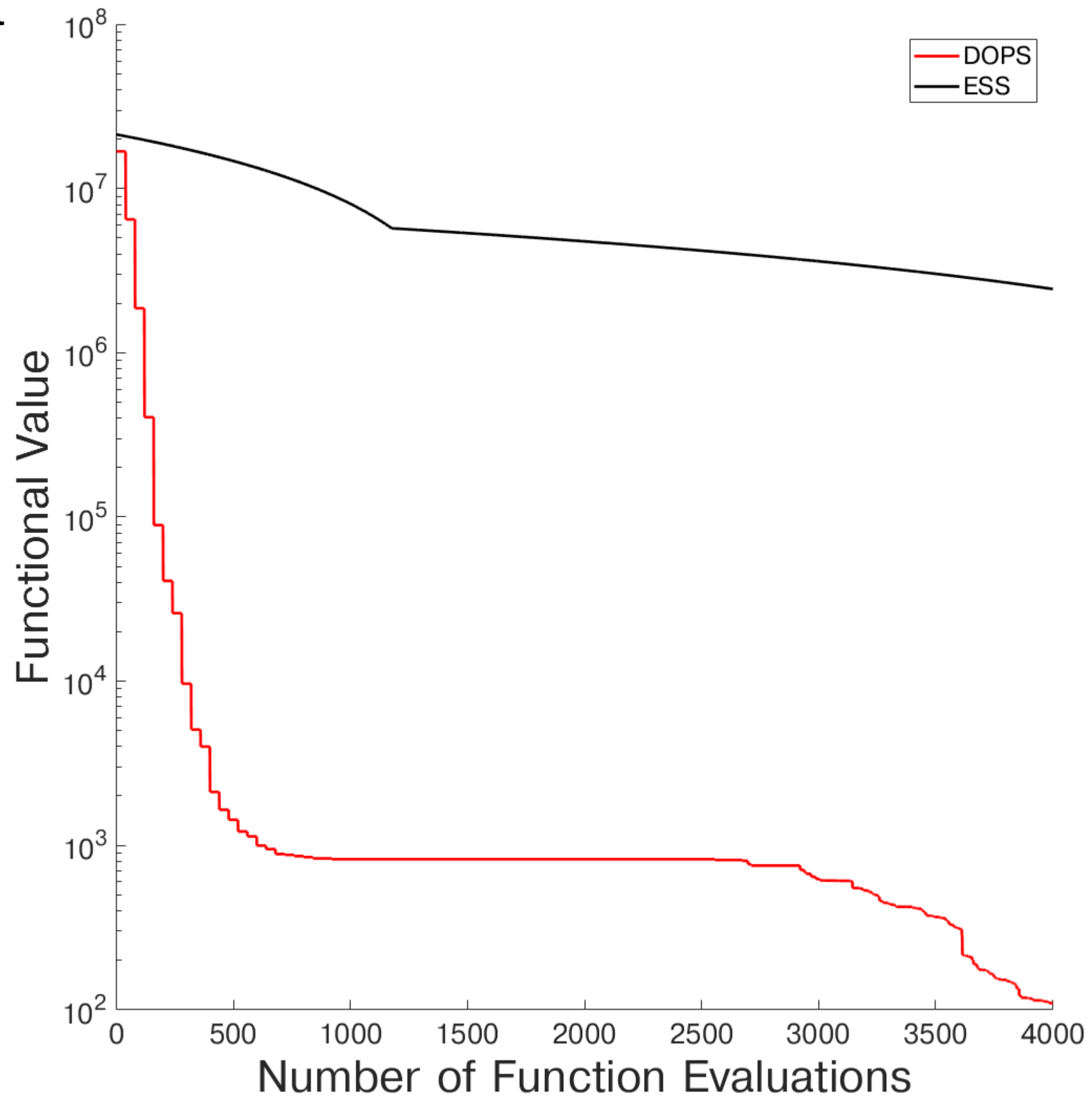

b

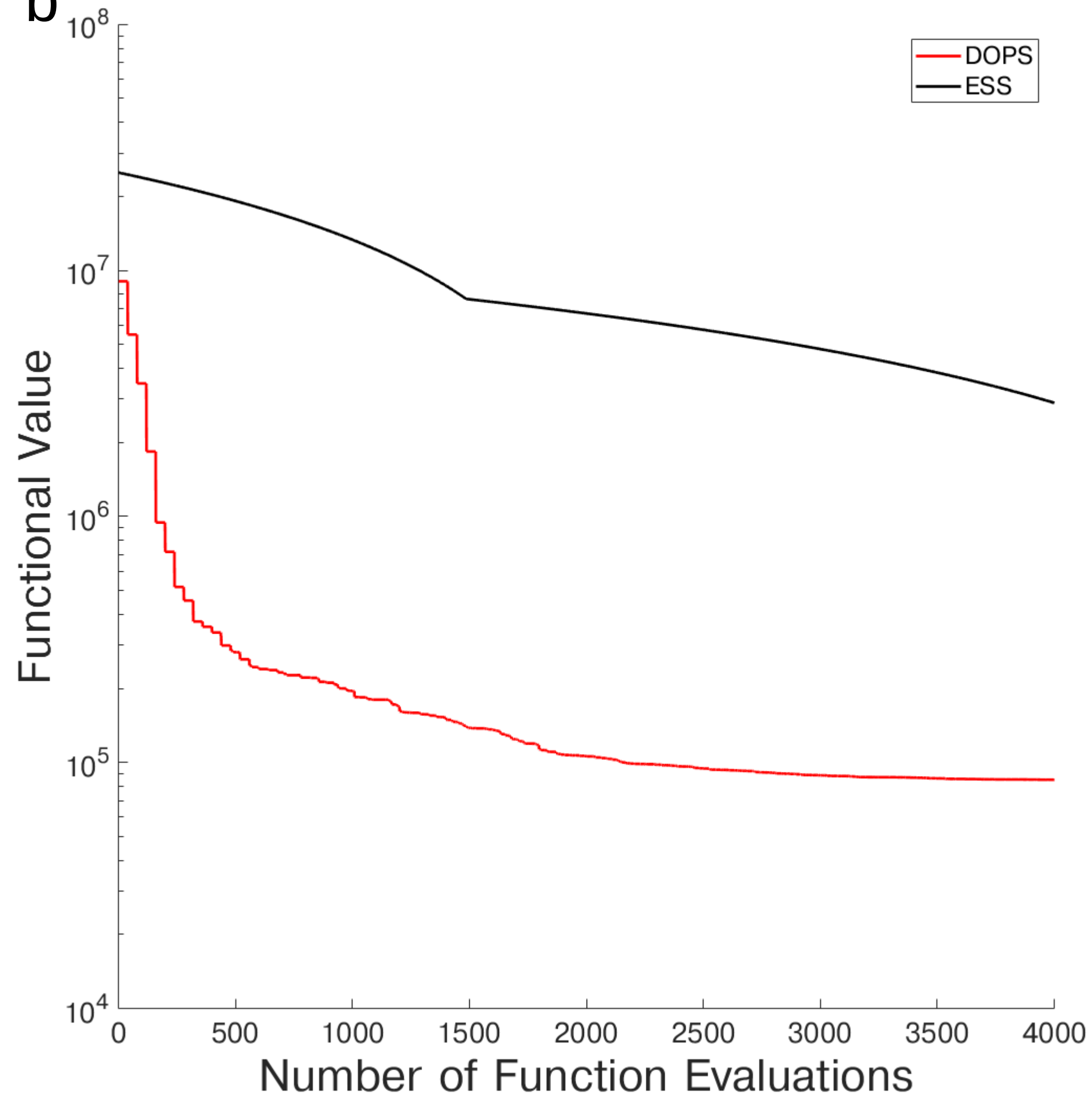

Supplement: Supplementary file 6 — Figure S6. Comparison of DOPS to ESS. (PDF 73 kb) [file 12918_2018_610_MOESM6_ESM.pdf]

**a**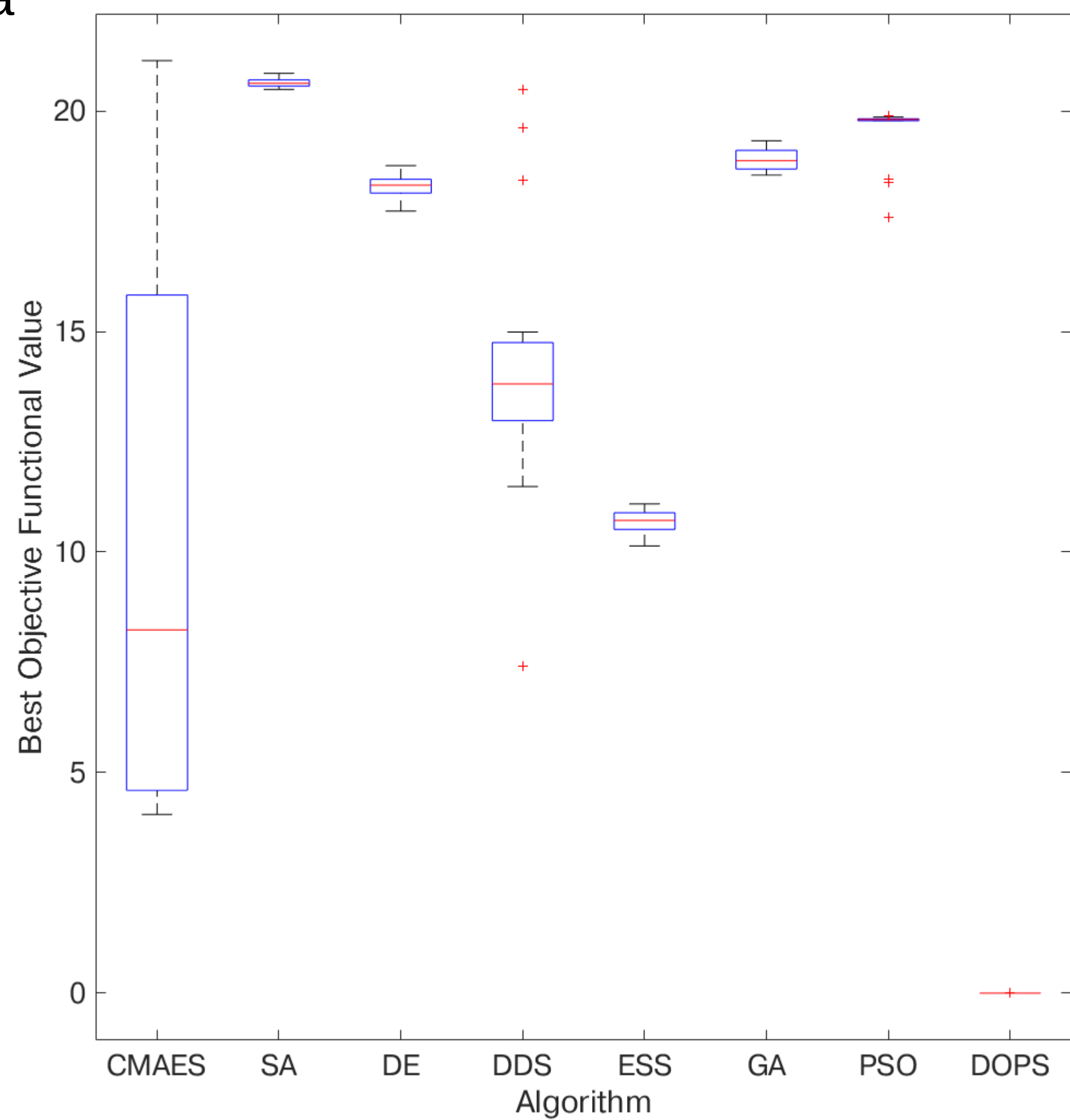**b**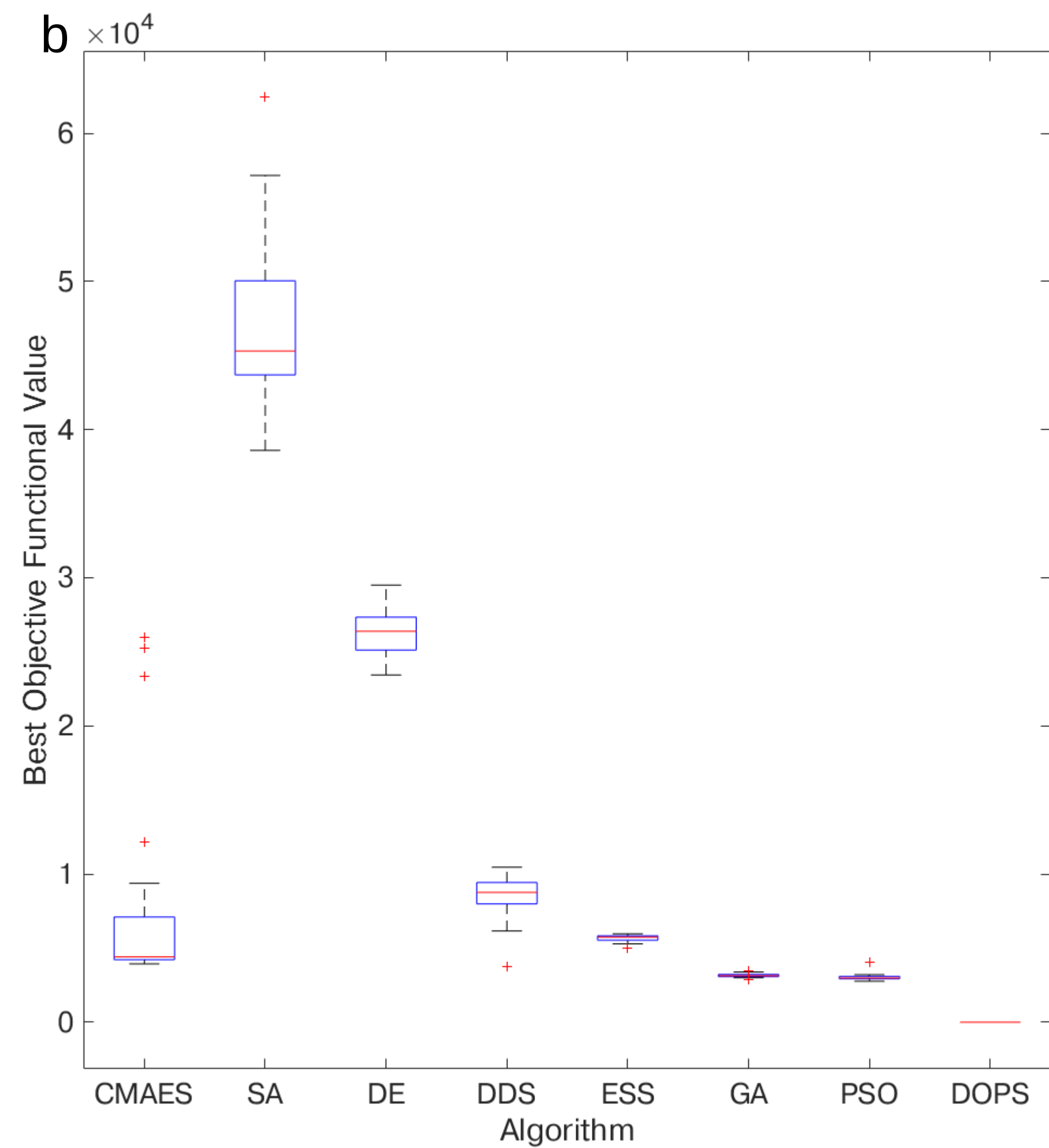**c**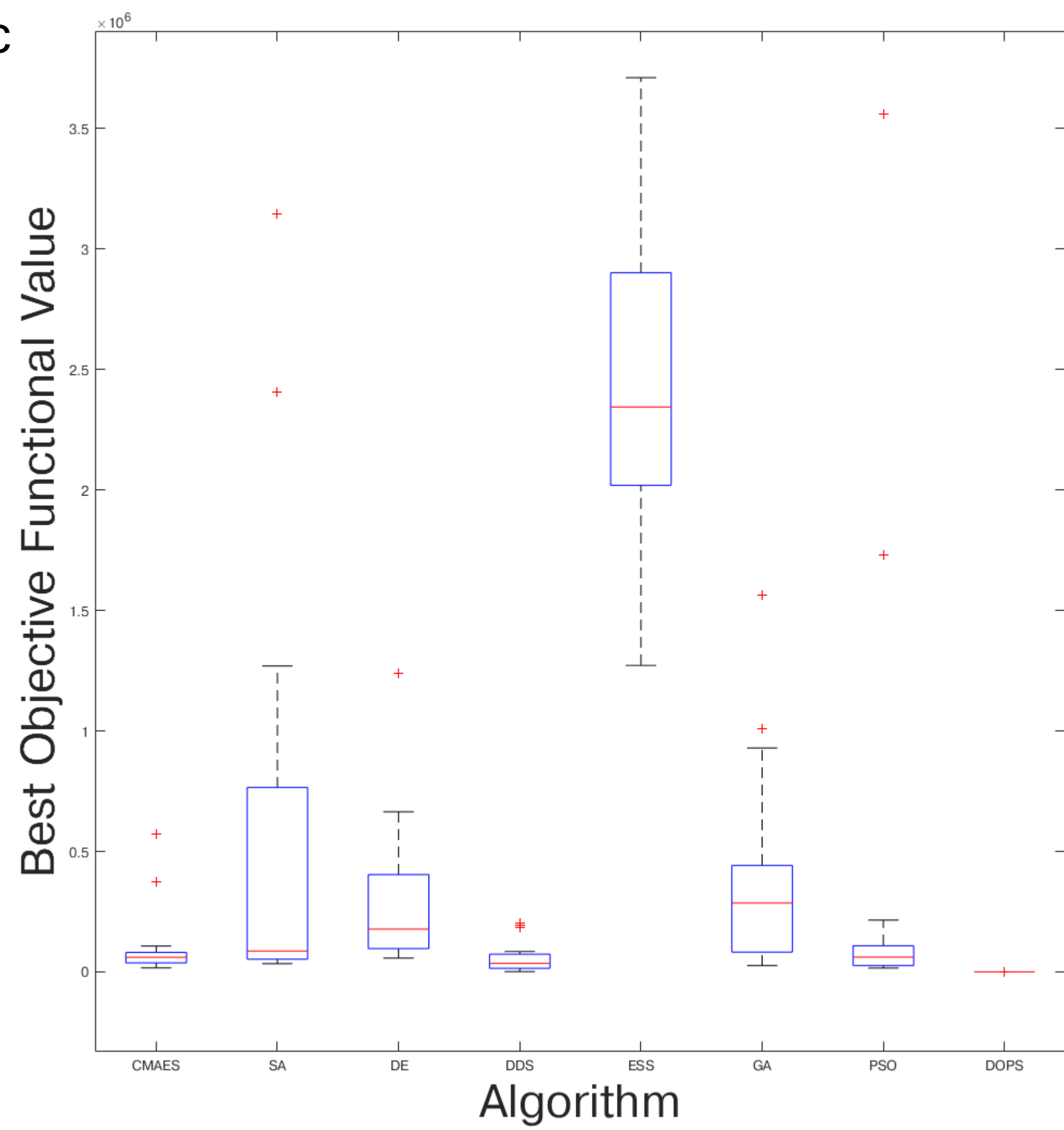**d**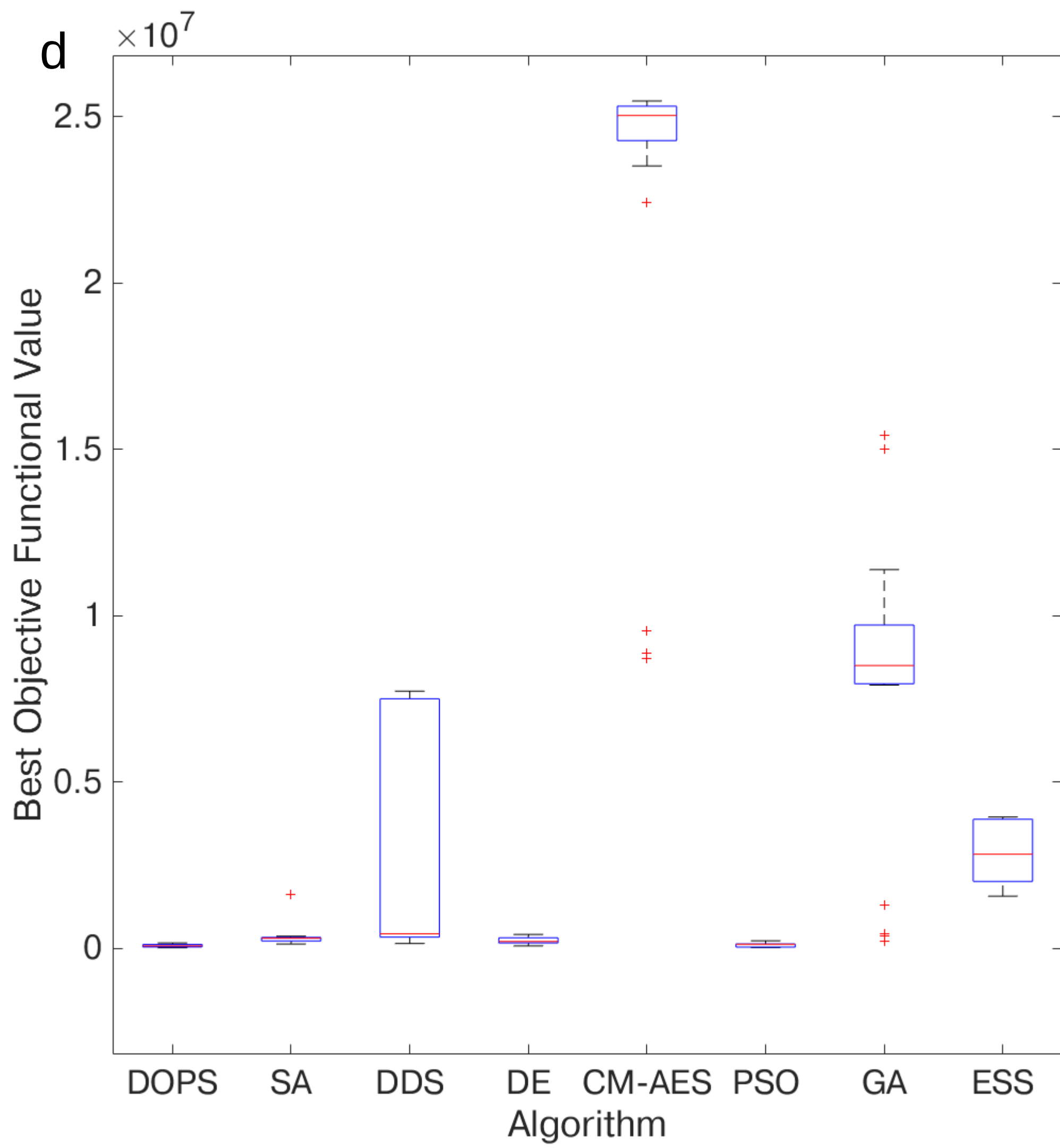

Supplement: Supplementary file 7 — Figure S7. Comparison of functional values. (PDF 123 kb) [file 12918_2018_610_MOESM7_ESM.pdf]

a

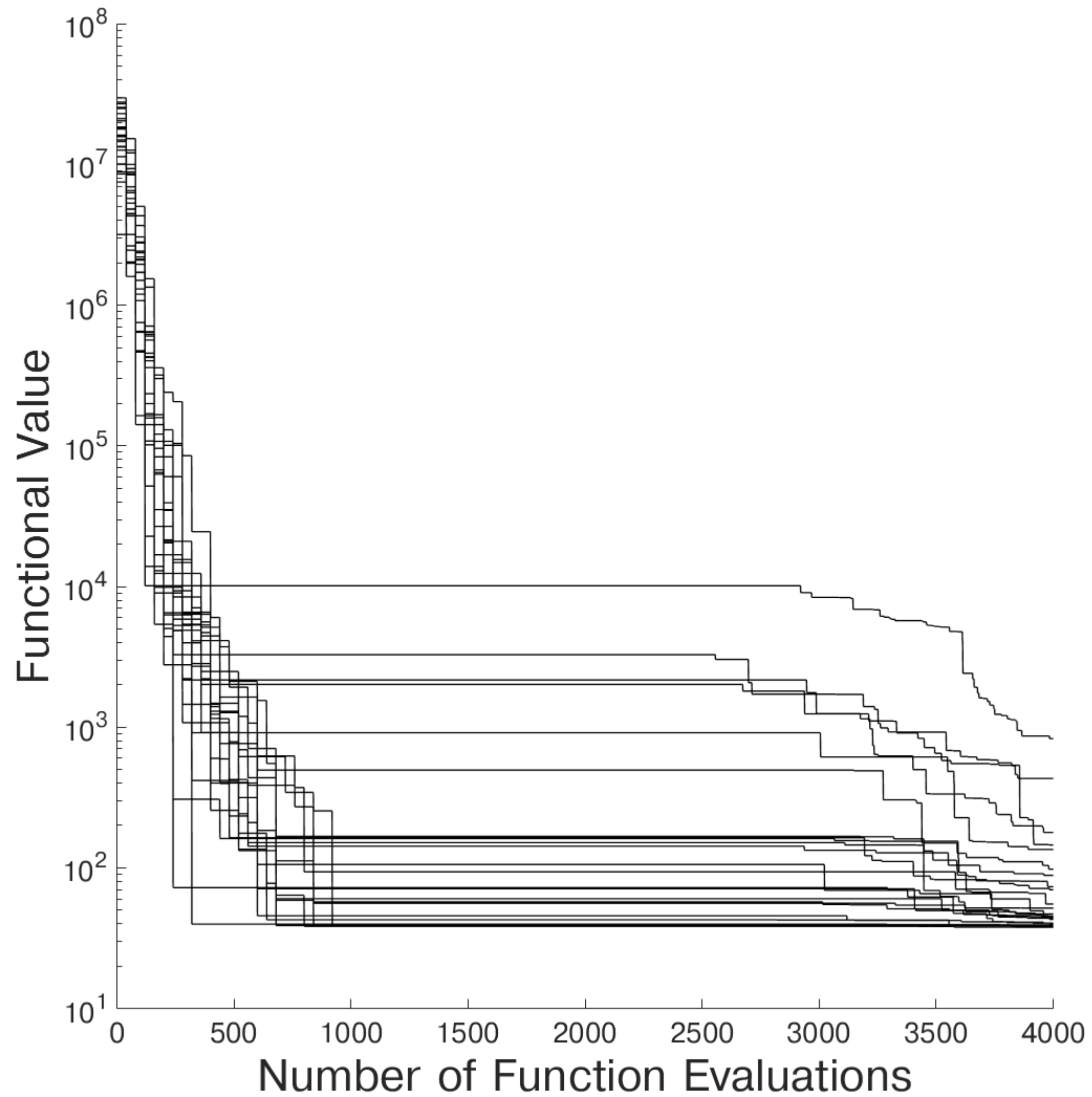

b

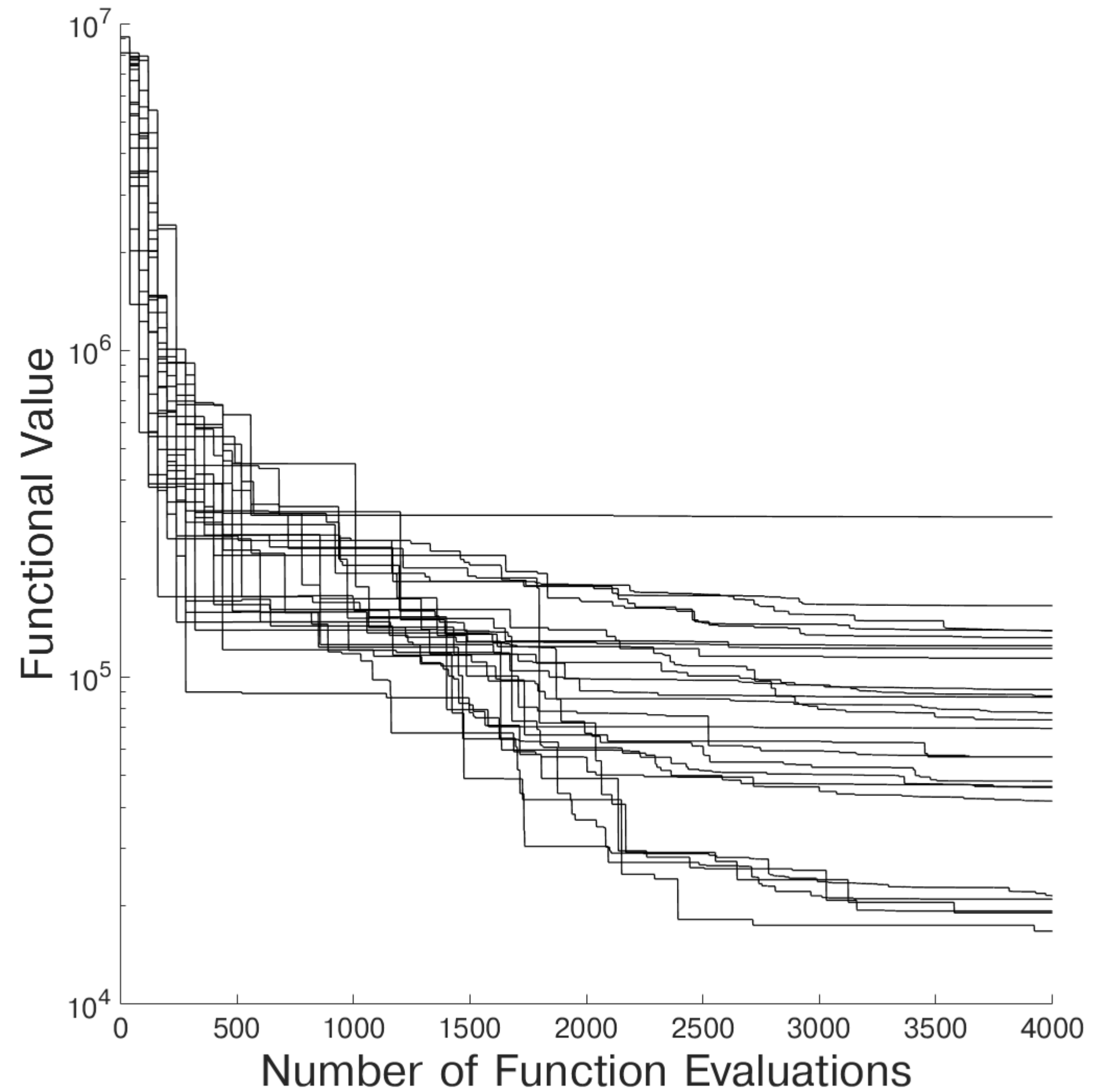

Supplement: Supplementary file 8 — Figure S8. Dispersion Curves. (PDF 89 kb) [file 12918_2018_610_MOESM8_ESM.pdf]
